# “Lobster cage” technique for transcatheter embolization of high-flow arteriovenous fistulas or portosystemic shunts

**DOI:** 10.1186/s42155-025-00538-x

**Published:** 2025-12-13

**Authors:** Wali Badar, Osman Ahmed, Faris Galambo, Wael Saad

**Affiliations:** 1 Division of Interventional Radiology, Department of Radiology, University of Illinois Hospital and Health Sciences System, Chicago, IL USA; 2 Division of Interventional Radiology, Department of Radiology, University of Chicago, Chicago, IL USA; 3 Division of Interventional Radiology, University of Utah School of Medicine, Salt Lake City, UT USA

Arteriovenous fistulas (AVF) and portosystemic shunts (PSS) present technical challenges during embolization given their size and high-flow pathophysiology [1]. Transcatheter embolization with coils and plugs for these entities have been well-described [2]. Their high-flow nature can however present challenges in coil deployment due to higher risk of coil migration and paradoxical embolization [3]. Additionally, the brisk flow can obscure more proximal branches due to the blood sump effect during angiography. A potential short landing zone can further contribute to technical challenges with precise deployment [4]. Furthermore, options for durable occlusion may be limited once an embolic device is placed within this landing zone and persistent flow is seen [5]. Herein, we aim to describe a “coils within a plug” technique utilized in two different clinical scenarios for safe and effective mechanical occlusion of high flow AVFs and PSSs. Institutional review board approval was not required for the following case presentations.

The first patient is a 30-year-old male with pancreatic transplant and pulmonary embolism (therapeutically anticoagulated for both) presented two days after transplant with suspected graft failure. Angiography demonstrated a moderate-sized, decompressing varix between the donor superior mesenteric artery and graft portal vein, compatible with an AVF (Fig. 1). Prior to embolizing the AVF, a balloon occlusion test was performed with the Berenstein balloon occlusion catheter (Boston Scientific, Marlborough, Massachusetts, USA) to determine the proximal most site of occlusion that would allow for patency of graft perfusing branches. A pancreaticoduodenal artery branch was identified near the takeoff of the AVF, exposing a shorter than originally anticipated landing zone to avoid exclusion of this graft perfusing vessel. Since the AVF contained brisk flow and the vessel landing zone was short in length, measuring 1.2 cm, plugs were chosen over coils to reduce migration risk. Two 5 mm Amplatzer-II vascular plugs (AVPs; St. Jude Medical,St. Paul, Minnesota, USA) were deployed within the AVF through a 5-French end hole Angle glide catheter (Terumo, Tokyo, Japan). On post-embolization angiography, sluggish but persistent AVF flow was noted. The procedure was terminated with the expectation that thrombosis would result. However, post procedurally graft function did not improve and Doppler ultrasound demonstrated a persistent AVF on ultrasound. The patient returned to interventional radiology the following day and angiography confirmed patent AVF with flow through the plugs, presumably secondary to inadequate clot formation within the nitinol mesh framework (Fig. 2). Subsequently, a 2.4-French Progreat (Terumo, Tokyo, Japan) microwire/microcatheter system were advanced into the interstices of the proximal AVP-II. Next, a 5-French end hole Kumpe catheter (Cook Medical, Bloomington, Indiana, USA) was telescoped over the microcatheter system into the proximal-most AVP, and multiple 0.018 and 0.035 Tornado coils (Cook Medical, Bloomington, Indiana) were tightly packed into the meshwork of the proximal AVP-II to “trap” the coils within the plug (Fig. 3). Follow up angiogram demonstrated complete stasis within the AVF (Fig. 4). Sequential 3- and 6-month transplant pancreas ultrasound exams showed complete stasis of the AVF with no evidence of recanalization.

**Fig. 1:**
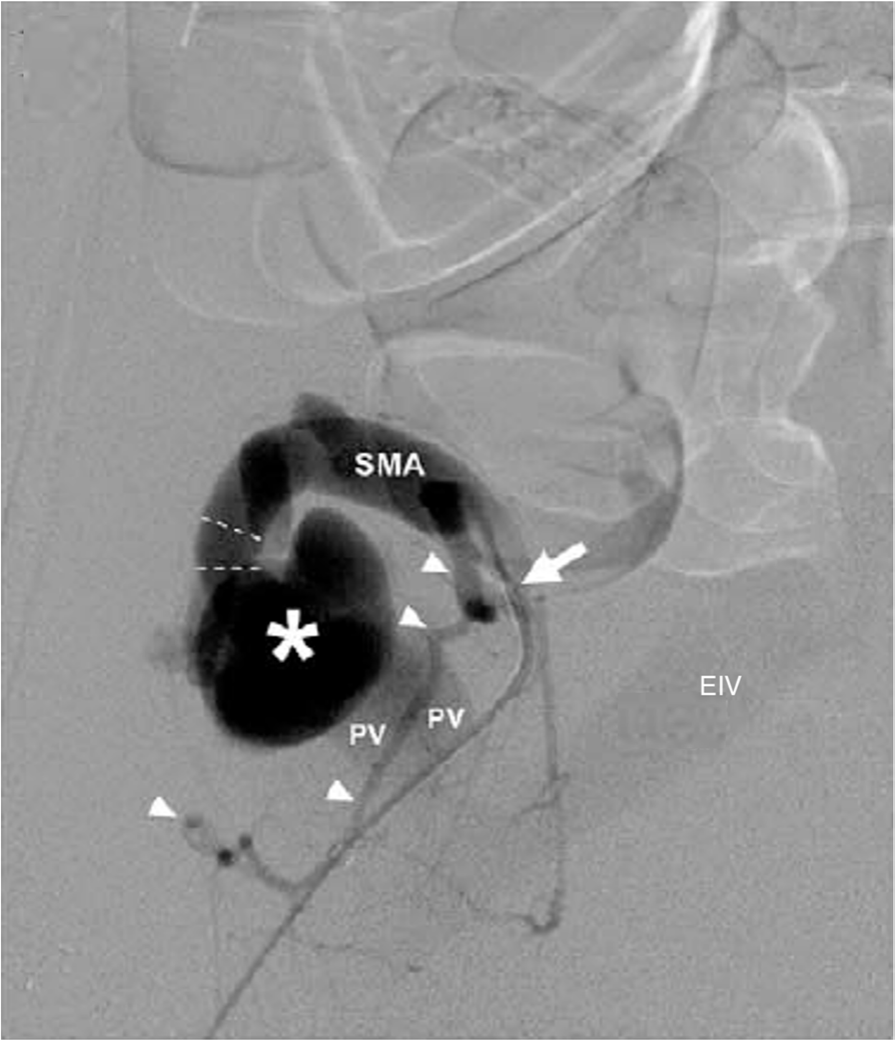
Arteriovenous fistula between host superior mesenteric artery and donor portal vein. Asterisk represents the fistula or decompressing varix which is accessed by a 5 French angle glide catheter (white arrow). Early filling of the external iliac vein is seen. Arrow heads demonstrate the pancreaticoduodenal artery. SMA = superior mesenteric artery; PV = portal vein; EIV: external iliac vein

**Fig. 2:**
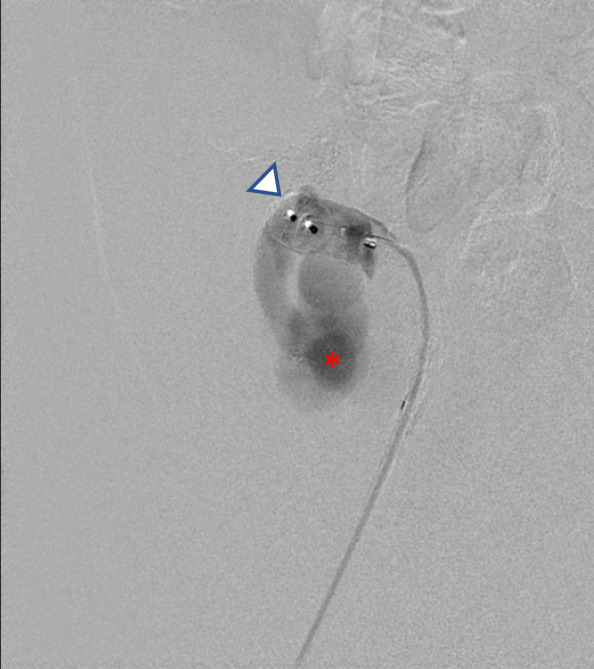
Persistent flow is seen through the Amplatzer plugs (arrowhead). Flow is seen through the varix (asterisk)

**Fig. 3:**
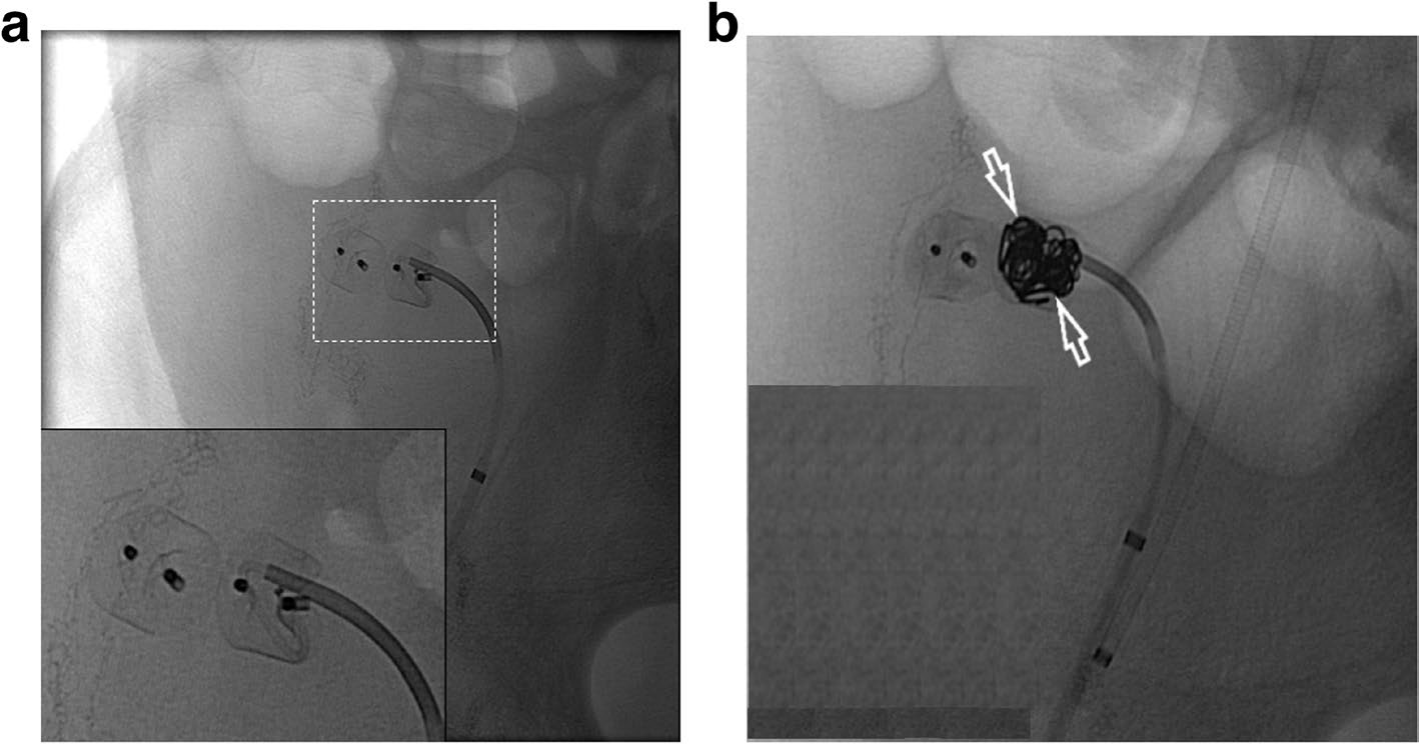
**a** 5 french kumpe catheter is seen advanced into the meshwork of the proximal Amplazter II plug. **b** Several 0.035 Tornado coils (white bordered arrows) have been packed into the proximal Amplatzer II plug

**Fig. 4:**
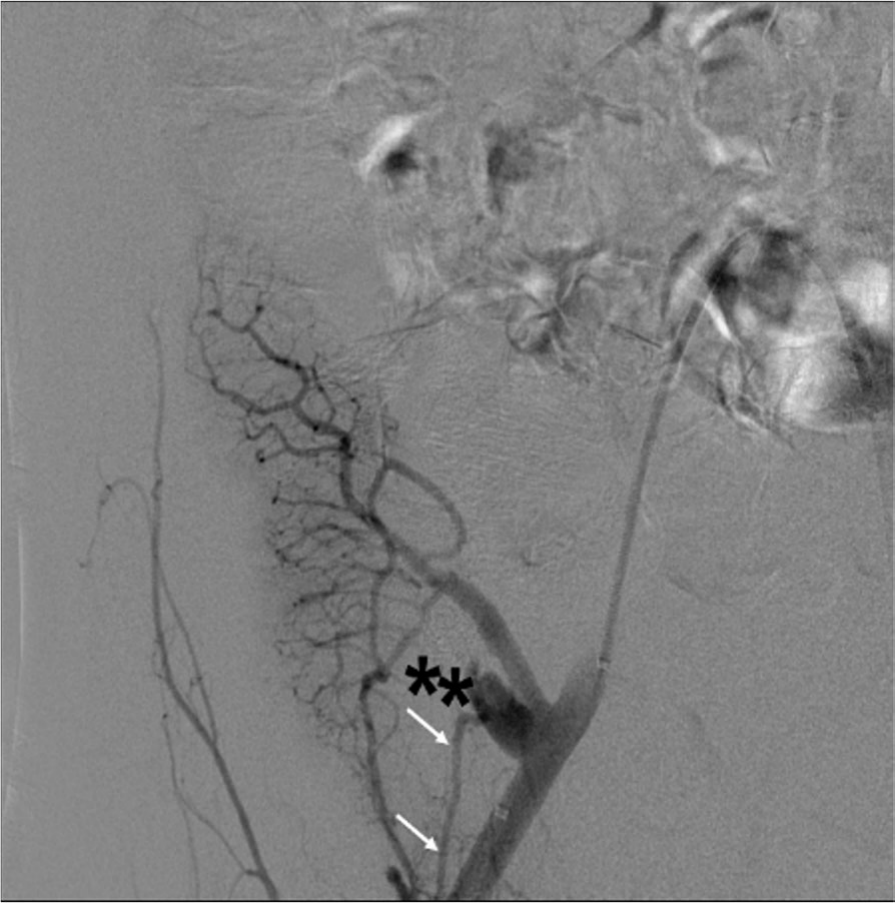
Complete stasis of flow within the arteriovenous fistula (**). A pancreaticoduodenal artery (white arrows) is noted

The second patient was a 61-year-old male with alcoholic cirrhosis, severe hepatic encephalopathy, portal vein thrombosis, and multiple PSSs undergoing liver transplant work-up. To optimize his transplant candidacy, he presented for transjugular intrahepatic portosystemic shunt (TIPS) creation, portal vein recanalization (PVR), and embolization of PSSs. A TIPS was placed via transjugular access. Portography demonstrated several PSSs with the largest from SMV to the right gonadal vein (Fig. 5). From a retrograde approach, two 10 mm AVP-II Plugs were placed in the efferent right gonadal vein through a 5 French angle glide catheter. Given the high-flow nature of this shunt and armed with the first patient’s experience, the decision was made to place fibered coils within the plug, similar to the first case. Given the favorable linear anatomy from the jugular vein access to the right gonadal vein sheath, a 21G 65 cm Chiba needle (Cook Medical, Bloomington, Indiana, USA), which was manually curved to provide directionality, was used to pierce the central nitinol mesh of the AVP-II. Directly through the needle, multiple 0.018″ nester coils (Cook Medical, Bloomington, Indiana, USA) were deployed (Fig. 6). Final portography demonstrated successful immediate occlusion of the shunt. Follow up 3-month triple phase CT showed complete resolution of the embolized portosystemic shunt with no evidence of recanalization.

**Fig. 5:**
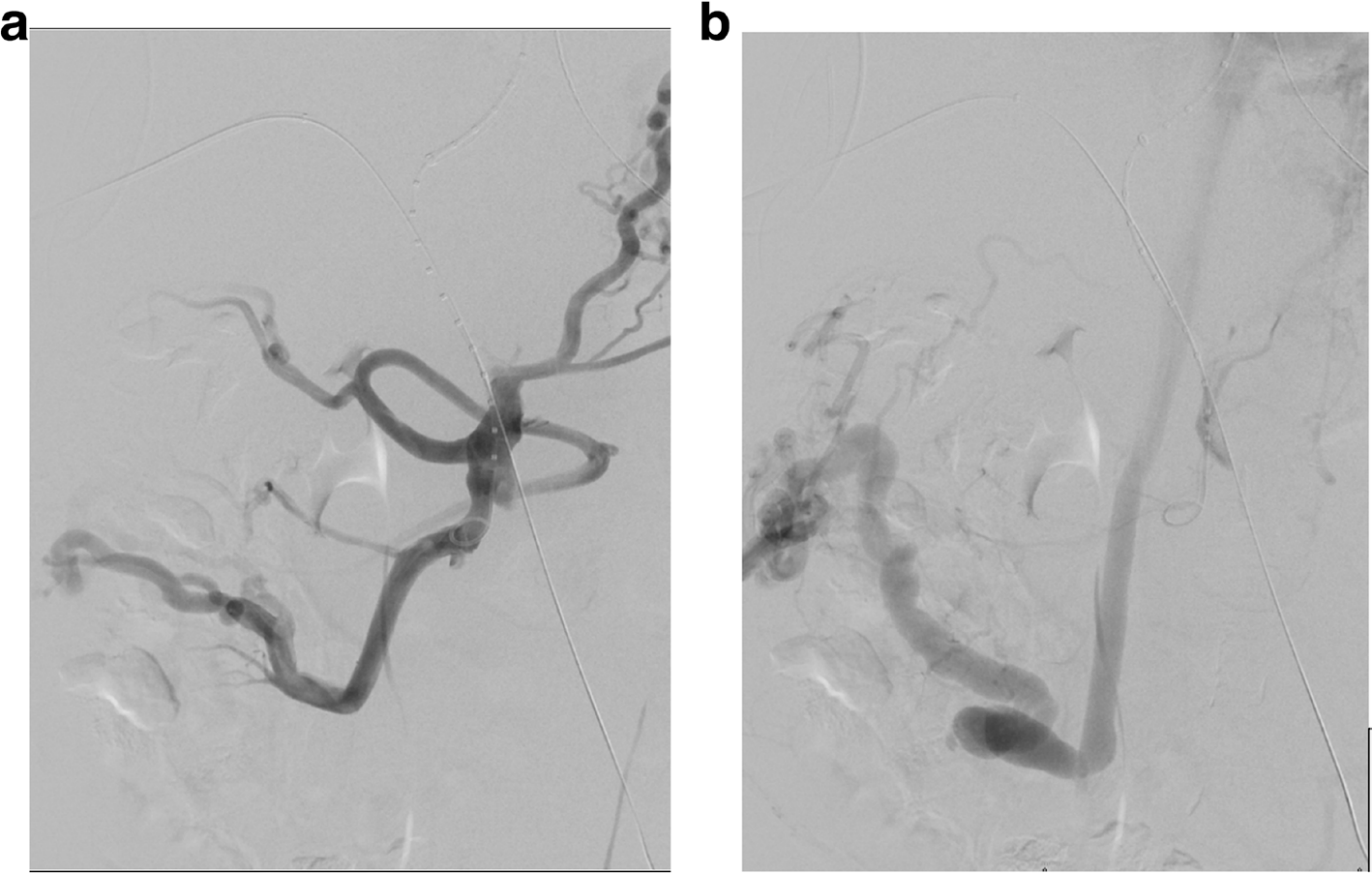
Early (**a**) and late (**b**) transhepatic portography from SMV near the portosplenic confluence demonstrating a large portosystemic shunt from SMV to right gonadal vein. Anatomy was deemed more favorable for retrograde occlusion

**Fig. 6:**
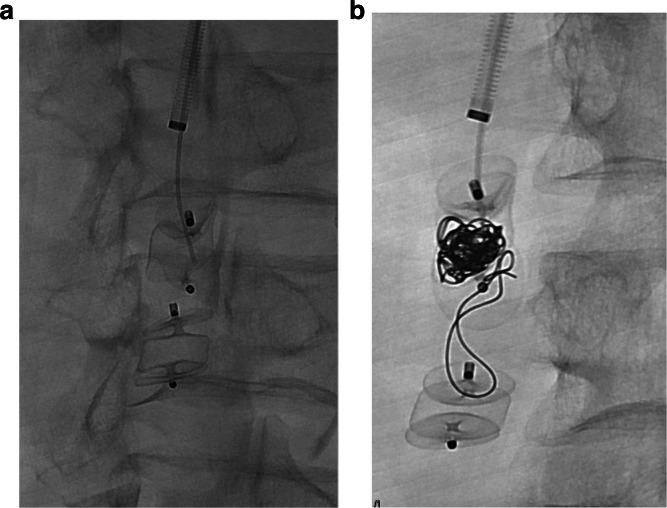
Image demonstrates needle cannulation of the central portion of the AVP-II’s nitinol mesh pre (**a**) and post (**b**) coil deployment. A single coil was inadvertently deployed between the two plugs rather than within the mesh

AVP failure due to persistent patency is relatively uncommon and usually occurs from under-sizing of the embolic device [6]. In the first case, persistent patency was likely due to therapeutic anticoagulation with possible contribution from the nearby pancreaticoduodenal artery and its branches (Fig. 1). The balloon occlusion test of an AVF can be performed to identify vessels that would otherwise be obscured by a high-flow AVF due to blood sumping and facilitate safe plug deployment [7]. Once these vessels are identified, plug/coil deployment can be planned to prevent inadvertent occlusion of these vessels due to suboptimal visualization. The above-described technique may serve as an endovascular bailout approach in the event of persistent patency with an appropriately-sized plug in a high-flow AVF and a short landing zone which may be seen in systemic AVFs, iatrogenic fistulas, dialysis access fistulas, and endoleaks. This method can alternatively be used primarily to facilitate immediate occlusion as was done in the second case. This “Lobster Trap” technique takes advantage of the rigid cage-like structure of the AVP-II, which allows for tight coil packing and immediate mechanical occlusion of the vessel without risk of coil migration or paradoxical embolism [8]. When performing this technique, it is important to consider risks associated with plug deployment such as plug displacement, from undersizing, or vascular perforation, from oversizing [9]. Coil deployment risks are also worthwhile to consider such as deployment outside of the plug’s framework which was seen in the second case (Fig. 6). This may have been due to use of the Chiba needle to pierce through the plug’s frame work resulting in coil deployment outside of the distal end of the proximal plug. Regardless, coil migration can be prevented by deploying two plugs as seen in case two. The technical feasibility of this intervention, through both catheter/microcatheter or needle deployment, adds to the versatility of the technique and in our opinion makes it worth sharing with practicing interventionalists.

## Data Availability

None.

## References

[1] Angle JF, Matsumoto AH, McGraw JK, Hagspiel KD, Spinosa DJ, McCullough CS. Percutaneous embolization of a high-flow pancreatic transplant arteriovenous fistula. Cardiovasc Intervent Radiol. 1999;22(2):147–9. 10.1007/BF03035357. PMID: 10094997. 10094997 10.1007/BF03035357

[2] Saad WE, Darwish WE, Turba UC, Angle JF, Wagner CE, Matsumoto AH, Brayman K, Hagspiel KD. Endovascular management of vascular complications in pancreatic transplants. Vasc Endovascular Surg. 2012;46(3):262–8. 10.1177/1538574412438949. Epub 2012 Apr 9 PMID: 22492112. 22492112 10.1177/1538574412438949

[3] Yetkin NA, Tutar N. Intravascular coil migration to bronchus: review of the literature with two case reports. Tuberk Toraks. 2019;67(4):307–13. 10.5578/tt.69010. English. PMID: 32050873. 32050873 10.5578/tt.69010

[4] Lopera JE. The amplatzer vascular plug: review of evolution and current applications. Semin Intervent Radiol. 2015;32(4):356–69. 10.1055/s-0035-1564810. PMID:26622098;PMCID:PMC4640916. 26622098 10.1055/s-0035-1564810 PMC4640916

[5] Maleux G, Rega F, Heye S, Troost E, Budts W. Asymptomatic migration of a first-generation AMPLATZER vascular plug into the abdominal aorta: conservative management may be an option. J Vasc Interv Radiol. 2011;22(4):569–70. 10.1016/j.jvir.2010.11.033. Epub 2011 Feb 12 PMID: 21316986. 21316986 10.1016/j.jvir.2010.11.033

[6] White HA, Travis SJ. The Amplatzer vascular plug. Cardiovasc Intervent Radiol. 2008;31(2):448–9. 10.1007/s00270-007-9259-3. Epub 2008 Jan 8. PMID: 18180988. 18180988 10.1007/s00270-007-9259-3

[7] Mansueto G, D’Onofrio M, Minniti S, Ferrara RM, Procacci C. Therapeutic embolization of idiopathic renal arteriovenous fistula using the “stop-flow” technique. J Endovasc Ther. 2001;8(2):210–5. 10.1177/152660280100800218. PMID: 11357984. 11357984 10.1177/152660280100800218

[8] Koganemaru M, Tanoue S, Kuhara A, Kugiyama T, Abe T. Internal coil packing method for the Amplatzer vascular plug 4. Diagn Interv Radiol. 2019;25(5):410–2. 10.5152/dir.2019.19009. PMID:31287427;PMCID:PMC6727819. 31287427 10.5152/dir.2019.19009 PMC6727819

[9] Pech M, Mohnike K, Wieners G, Seidensticker R, Seidensticker M, Zapasnik A, Ricke J, Dudeck O. Advantages and disadvantages of the Amplatzer Vascular Plug IV in visceral embolization: report of 50 placements. Cardiovasc Intervent Radiol. 2011;34(5):1069–73. 10.1007/s00270-011-0150-x. Epub 2011 Mar 24 PMID: 21431965. 21431965 10.1007/s00270-011-0150-x

